# Concomitant Use of Transcranial Direct Current Stimulation and Computer-Assisted Training for the Rehabilitation of Attention in Traumatic Brain Injured Patients: Behavioral and Neuroimaging Results

**DOI:** 10.3389/fnbeh.2016.00057

**Published:** 2016-03-31

**Authors:** Katiuscia Sacco, Valentina Galetto, Danilo Dimitri, Elisabetta Geda, Francesca Perotti, Marina Zettin, Giuliano C. Geminiani

**Affiliations:** ^1^Imaging and Cerebral Plasticity Research Group, Department of Psychology, University of TurinTurin, Italy; ^2^Neuroscience Institute of Turin, University of TurinTurin, Italy; ^3^Centro PuzzleTurin, Italy

**Keywords:** transcranial direct current stimulation (tDCS), cognitive rehabilitation, attention, traumatic brain injury (TBI), functional magnetic resonance imaging (fMRI), cerebral plasticity

## Abstract

Divided attention (DA), the ability to distribute cognitive resources among two or more simultaneous tasks, may be severely compromised after traumatic brain injury (TBI), resulting in problems with numerous activities involved with daily living. So far, no research has investigated whether the use of non-invasive brain stimulation associated with neuropsychological rehabilitation might contribute to the recovery of such cognitive function. The main purpose of this study was to assess the effectiveness of 10 transcranial direct current stimulation (tDCS) sessions combined with computer-assisted training; it also intended to explore the neural modifications induced by the treatment. Thirty-two patients with severe TBI participated in the study: 16 were part of the experimental group, and 16 part of the control group. The treatment included 20’ of tDCS, administered twice a day for 5 days. The electrodes were placed on the dorso-lateral prefrontal cortex. Their location varied across patients and it depended on each participant’s specific area of damage. The control group received sham tDCS. After each tDCS session, the patient received computer-assisted cognitive training on DA for 40’. The results showed that the experimental group significantly improved in DA performance between pre- and post-treatment, showing faster reaction times (RTs), and fewer omissions. No improvement was detected between the baseline assessment (i.e., 1 month before treatment) and the pre-training assessment, or within the control group. Functional magnetic resonance imaging (fMRI) data, obtained on the experimental group during a DA task, showed post-treatment lower cerebral activations in the right superior temporal gyrus (BA 42), right and left middle frontal gyrus (BA 6), right postcentral gyrus (BA 3) and left inferior frontal gyrus (BA 9). We interpreted such neural changes as normalization of previously abnormal hyperactivations.

## Introduction

Traumatic brain injury (TBI) is one of the most common causes of disability and social withdrawal in young individuals (Andelic et al., 2009). These patients typically require long-lasting and costly rehabilitation; the outcome, depending on many factors, may vary among cases. Rehabilitation research should, on the one hand, design and test the effectiveness of innovative rehabilitation strategies and on the other, identify how patients can benefit from different rehabilitation programs in order to maximize the individual chances of improvement. This is particularly important in the case of TBI patients, as their cerebral lesions and the neurobehavioral consequences vary greatly from one person to another.

Among the cognitive changes that may occur following TBI, the most prominent are divided attention (DA) and memory disorders (Asloun et al., 2008; Cyr et al., 2009). Indeed, patients, their caregivers and rehabilitation professionals frequently report their difficulty in doing two things simultaneously, and this difficulty may negatively impact on their return to work (van Zomeren and van den Burg, 1985; Ponsford and Clements, 1991; Couillet et al., 2010). Research on DA in patients with TBI has shown that they do not have any difficulty when the DA tasks can be carried out relatively automatically. On the contrary, they are impaired when the exercise becomes more complex or it needs to be performed under high time-pressure, requiring considerable working memory load or executive control (Park et al., 1999; Leclercq and Azouvi, 2002). For example, in the study conducted by Park et al. (1999) severe TBI patients and control subjects were asked to perform two tasks involving working memory. The exercises had to be executed first separately and then concurrently. The results showed that the TBI group had significantly more difficulties when performing the two tasks concurrently, although their performance does not statistically differ from that of healthy participants when the same tasks were done separately. In other studies involving patients with severe TBI, deficits in DA have only been found in the more demanding conditions (Brouwer et al., 2001, 2002). Most importantly, dual-task measures under the most difficult conditions seem to be strictly correlated with performance in daily-living activities, thus suggesting the key role of DA in everyday life (Withaar et al., 2000). Similar results were reported by Azouvi et al. (2004), who assessed dual-task performance in TBI patients, under three different experimental conditions, in which the participants were asked to pay equal attention to both tasks or to focus mostly on one of them. TBI patients only showed a disproportionate increase in reaction times (RTs) under the dual-task condition. All these results considered, DA should be one of the main targets in the rehabilitation of TBI patients, given its key role in many tasks of everyday life (Toyokura et al., 2012; Masson et al., 2013). However, few rehabilitation programs have been developed to improve this function in patients with TBI [(Couillet et al., 2010) specifically addressed DA; (Serino et al., 2007; Montani et al., 2014) included it in their training programs]. More generally, to date there is limited evidence of the effectiveness of cognitive rehabilitation programs aimed at improving attention (Cernich et al., 2010; Cicerone et al., 2011). Innovative rehabilitation strategies in this field are thus needed.

In this article, we present a training program for the rehabilitation of DA. We used a computer-based procedure: it has been demonstrated that the use of computer-assisted training leads to greater motivation, better training performance and better performance at post-training than using regular training (De Luca et al., 2014). A further innovative aspect of our trial was the use of neurostimulation procedures, which preceded every treatment session, in order to prepare the brain for the following exercises. Neurostimulation strategies using various forms of electrical stimulation have recently been applied to intervene on functional deficits in animal models and clinical trials. The main outcomes of these sets of research suggest a key role of neurostimulation in enhancing both motor and cognitive deficits after brain injury (Miniussi et al., 2008; Shin et al., 2015). Indeed, modifying cortical excitability may lead to a more functional neural reorganization (Villamar et al., 2012). Such techniques, aimed at boosting neuroplastic changes in the brain, are expected to enhance the effects of behavioral interventions in neurological diseases (Schulz et al., 2013; Clark and Parasuraman, 2014): cortical electrical stimulation combined with appropriate cognitive training may facilitate cerebral reorganization and consolidation of learning in the specifically trained neural networks. Among non-invasive brain stimulation techniques, transcranial direct current stimulation (tDCS) has recently gained consideration as it has shown promise in the enhancement of motor and cognitive functions (Flöel, 2014). It involves passing a direct electrical current through the cerebral cortex via electrodes placed upon the scalp. Although the passage of the electrical signal in the overlying tissues entails a partial current dispersion, the stimulus that reaches the brain is intense enough to change the resting membrane potential, thus modifying the level of spontaneous neuronal excitability. tDCS serves as a neuromodulatory intervention: our rehabilitation protocol, involving the concomitant use of tDCS and computer-assisted exercises in DA, is aimed at modulating neural plasticity to improve performance. We applied tDCS over the dorsolateral prefrontal cortex (DLPFC), given its essential role in bimodal DA. Indeed, many studies (Johnson and Zatorre, 2005, 2006; Wagner et al., 2006; Johnson et al., 2007) have demonstrated that dividing attention between simultaneous auditory and visual events leads to increased activity in the DLPFC. This area seems to be causally involved in DA: in a research by Johnson et al. (2007), it was demonstrated that inhibiting the activity of the left posterior DLPFC through slow rTMS induced a significant decrease of DA in most of the participants.

In order to evaluate the effectiveness of this rehabilitation program, we collected pre- and post-treatment behavioral assessment measures; we also used functional magnetic resonance imaging (fMRI) to monitor possible changes following the therapeutic intervention, which may unravel mechanisms of treatment-related neuroplasticity. We expect that the concomitant use of tDCS and a computerized training is more effective in improving the DA ability than the use of a computerized training alone. Also, we predict that such improvements are accompanied by cerebral modifications within networks involved in dual task processing: fronto-parietal regions have been identified to be specifically involved in processing more information at a time (Herath et al., 2001; Collette et al., 2005; Nebel et al., 2005), with the inferior and middle frontal gyri holding a crucial role (for a review, see Klingberg, 2000).

## Materials and Methods

### Participants

Thirty-two adult patients with TBI completed the rehabilitation program: 16 patients (4 females and 12 males) participated as part of the experimental group, and 16 patients (2 female and 14 males) participated as part of the control group. Participants were randomly assigned to the experimental group or to the control one. The experimental group ranged in age from 18 to 65 years (*M* = 37.7; *SD* = 10.4); their level of education ranged from 8 to 18 years of schooling (*M* = 11.5; *SD* = 3.48). The control group ranged in age from 18 to 66 (*M* = 35,2; *SD* = 12,9), and their education level was comprised between 5 and 17 (*M* = 10,5; *SD* = 4,09). The two groups did not statistically differ with respect to age (*t* = −0.488; *p* = 0.629) and educational level (*t* = −0.816; *p* = 0.421). Participants were recruited at the Centro Puzzle, a local rehabilitation center for patients with severe brain injury. The time after onset ranged from 3.16 to 17.5 years (*M* = 8.73, *SD* = 4.45). All of the patients were victims of severe TBI: the Glasgow Coma Scale in the acute phase had been equal to or less than 8. The majority of the patients had sustained their injury in a road traffic accident. At the time of the study, all the patients were living at home, and none of them lived independently without their partners or parents.

Inclusion criteria for the study were the following: patients had to (1) be at least 18 years old; (2) be at least in their 12th month after the brain injury, in order to be sure that their cognitive profile was stable; (3) be Italian native speakers; (4) be in possession of adequate cognitive skills and comprehension abilities, certified by the achievement of a cut-off score of 24/30 on the Mini Mental State Examination (MMSE; Folstein et al., 1975) and of 29/36 on the Token Test (De Renzi and Vignolo, 1962). Exclusion criteria were the following: (1) prior history of TBI or other neurological disease; (2) neuropsychiatric illness; (3) pre-morbid alcohol or drug addiction, evaluated on the basis of the anamnestic data; (4) metal objects in the body such as aneurysm and hemostatic clips, implanted electrodes and electrical devices such as pacemakers, orthopedic material and devices. All of the participants gave their written informed consent to participate in the research. Approval for the study had previously been obtained from the local ethics committee (Centro Puzzle, protocol no. 1/2015).

### Experimental Design and Behavioral Measures

**Figure 1:**
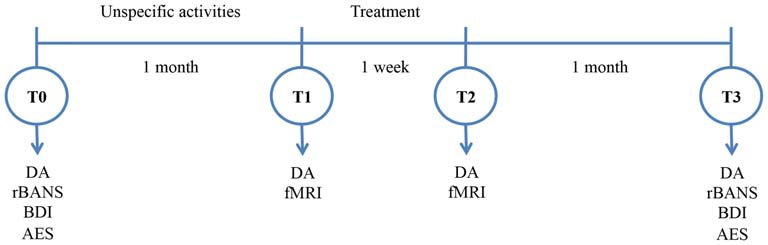
**Experimental design.** DA, Divided Attention; RBANS, Repeatable Battery for the Assessment of the Neuropsychological Status; BDI, Beck’s Depression Inventory; AES, Apathy Evaluation Scale.

The study was conducted over a period of 9 weeks, comprising a 5-day period of treatment and four assessment sessions (see Figure 1).

#### T0_Baseline

One month before the beginning of the treatment, the attentional abilities of the recruited patients were assessed using the DA subtest of the Test for the Examination of Attention (TEA; Zimmermann and Fimm, 2002). It consisted of a visual and an auditory computer-based task, which had to be performed simultaneously. Participants were asked to look at patterns of stimuli (letter X) moving on a computer screen, and to press a key only when they were in a specific arrangement. At the same time, they listened to high and low pitched sounds, and had to press a button only when the same pitch was repeated twice in succession. Moreover, the Repeatable Battery for the Assessment of the Neuropsychological Status (RBANS; Randolph et al., 1998) comprises tests of visual-spatial abilities, semantic fluency, selected attention, working memory and long-term memory, and it was administered to provide an evaluation of patients’ abilities in different cognitive domains. Finally, the Beck’s Depression Inventory (BDI; Beck et al., 1961) and the Apathy Evaluation Scale (AES; Marin et al., 1991) were administered to investigate whether a possible improvement of attention abilities might result in an enhancement of the affective state and therefore, in a significant reduction of depression and apathy. After the assessment, patients attended two sessions a week, for 1 month, during which they engaged in activities which were not specifically focused on improving attention. These included socializing activities, such as group recreation and games, as well as intellectual and creative activities, like reading newspapers, cooking and painting. The aim of this procedure was to detect any improvements in patients’ attentional abilities due to spontaneous recovery, or as a consequence of unspecific activities, or of simply taking part in a research program.

#### T1_Pre-Treatment

A few days before the beginning of the treatment, the patients’ attentional abilities were re-assessed with the DA subtest of the TEA, in order to verify the absence of any improvement due to attending sessions of unspecific activities between T0 and T1. Patients belonging to the experimental group also underwent the pre-treatment fMRI examination while performing a DA task.

#### T2_Post-Treatment

Immediately after the end of the treatment, the DA subtest of the TEA was administered to the participants for the third time, in order to prove the efficacy of the treatment on their attentional performance. Patients belonging to the experimental group also underwent the post-treatment fMRI examination to evaluate the changes that had occurred.

#### T3_FollowUp

One month after the end of the rehabilitation program the patients’ attentional and cognitive abilities were reassessed using the RBANS and the DA subtest of TEA, to evaluate the stability of their improvements in time. The BDI and AES were administered for the second time.

In order to rule out learning effects, in the DA test stimuli appeared randomly on the computer screen, and parallel versions of the RBANS were used at the different time points.

### fMRI Task

In addition to the behavioral assessment, patients underwent an fMRI scan before and after the treatment, in order to evaluate any changes in neuronal activity attributable to the treatment. Data acquisition was performed at the Koelliker Hospital in Turin, on a 1.5-T Philips Intera with a Sense high field high resolution head coil optimized for functional imaging. Patients with anxiety disorders such as claustrophobia, panic attacks or any disorder which could be severely exacerbated by confined spaces were excluded. A total of 11 patients completed the exam. In case of an emergency, the patients could press a button in order to ask for help and if necessary, interrupt the procedure at any time.

Functional areas of activations were assessed with a crossmodal DA task (Jackson et al., 2011). The task involved visual and auditory stimuli presented in an epoch-based design. During the task, visual stimuli (the letters “M” and “W” in white font on black background) were projected onto a screen using an LCD projector, and the images were viewed via a mirror positioned approximately 11 cm above the subject’s eyes on the head coil. Auditory stimuli were played through headphones (two sinusoidal pure tones, 1500 Hz and 500 Hz, 16 bit stereo at 44.1 kHz). Each stimulus was presented for 300 ms with a variable interstimulus interval of between 400 ms and 1000 ms. The task consisted of four conditions (baseline, visual selective attention (VA), auditory selective attention, and DA) each lasting for five acquisition volumes (16 s) that were repeated eight times in a random sequence. For each subject, a stimulus for each of the visual and auditory modalities was randomly selected as the target stimulus (or stimuli) for each condition. Targets were presented with a 20% probability, and the subjects’ task was to press a corresponding response button on an MR compatible keypad that they held in their dominant hand. Subjects were instructed to fixate on a cross hair on a screen in front of them during all different epochs. At the start of each block, instructions were displayed on the screen for 4 s: for baseline epochs subjects passively observed the sounds and letters; for VA selective epochs, subjects pressed the right response button every time their visual target (either an M or a W) appeared on the screen and ignored the tones; for auditory selective attention (AA) epochs, subjects pressed the left response button when they heard their target tone (either a 1500 Hz or 500 Hz tone) and ignored the letters; and for DA epochs, subjects pressed the right button when the target letter appeared on the screen, and the left button when the target tone was heard. A rest period, during which no task was presented, was given halfway through the task for eight volumes (25.6 s). A practice session was completed in a mock scanner before each session to minimize practice effects, and to familiarize subjects with the scanning environment. Finally, a set of anatomical MRI images were acquired (10 min).

### Treatment

The treatment consisted of 10 sessions. each session included 20 min of tDCS stimulation followed by 30 min of DA training. The control group received 25 s of tDCS stimulation, then the device turned off automatically (sham conditions). The treatment was provided in two sessions per day and lasted 5 days (for recommended number of sessions and tDCS treatment duration see Scelzo et al., 2011). Each session lasted approximately 1 h. Both electrode montage and rehabilitation activities were performed by a trained psychologist, who was assisted by a neurologist, to intervene if and when necessary. The experiment was conducted in a quiet room, in the rehabilitation center where the participants were recruited. The treatment focused on DA: all the activities were designed to increase participants’ ability to perform two tasks simultaneously. Specifically, they were asked to pay attention to auditory stimuli while performing VA tasks.

The tDCS stimulation was performed using a HDCstim device (Newronika srl, Milan, Italy). The electrodes (5 × 7 cm) were placed over the DLPFC, given its essential role in bimodal DA (Wagner et al., 2006; Johnson et al., 2007). However, the tDCS site differed between patients depending on the area of lesion, determined on the basis of the anatomical MRI scan. For those who had been excluded from the neuroimaging exam the electrode placement was established through the consultation of their clinical history and their previous scans. Overall, a dual channel stimulation (two anodes, one on the right DLPFC and the other on the left DLPFC, earth on the arm) was used with six patients having an equal hemispheric lesion distribution, while a mono channel stimulation was used with ten patients having unilateral lesions (anode on the lesioned hemisphere and cathode on the other hemisphere). In both cases the current intensity was 2 mA and its density 0.057 mA/cm^2^. Both these indexes were kept below the safety limits (Poreisz et al., 2007). Participants’ tolerance of the stimulation was constantly monitored. Furthermore, at the end of the procedure, each subject was invited to fill out a brief questionnaire on the adverse effects of the stimulation.

### Statistical Analysis

Paired *t*-test analyses were conducted in order to evaluate improvements between baseline and pre-training (where we expected no difference) and between pre-training and post-training (where we expected significant differences in performance on the DA task within the experimental but not within the control group). Paired *t*-tests on the other tasks’ measures were computed with the aim of exploring whether changes in other cognitive domains took place after training. A split-plot ANOVA was used in order to evaluate the interaction between Time (assessment phase) and the type of group (experimental vs. control), taking into account age, level of education and severity of lesion as covariates. Level of significance was chosen to be *p* < 0.05.

The fMRI data were analyzed using Brain Voyager QX Software (Brain Innovation, Maastricht, Holland). The functional data of each subject were pre-processed as follows: (1) Mean intensity adjustment in order to prevent global signal variability; (2) slice scan time correction, through the use of a sinc interpolation algorithm; (3) 3D motion correction: all the volumes were spatially aligned to the first volume by rigid body transformations, adopting a algorithm of trilinear interpolation; (4) spatial smoothing with a Gaussian kernel of 4 mm FWHM; and (5) temporal filters, i.e., linear and non-linear trend removal through the use of a temporal high-pass filter (frequency pass = 0.008 Hz), adopted to remove drifts due to scanner and other low frequency noises. After pre-processing, the temporal series of each voxel were filtered using a band-pass filter (0.01 < *f* < 0.08 Hz) in order to remove both the very low frequencies and the noise due to high frequencies (respiratory and cardiac frequencies). The filtered time series was then transformed into a frequency domain using Fourier transformation; this process allowed us to decompose a signal made up of several frequencies and identify the spectrum of the signal. The power spectrum represents the energy of the signal at different frequencies. Then a statistical analysis using the General Linear Model was performed to yield functional activation maps during the pre- and post-tests separately. Subsequently, the General Linear Model was used to compare post-test activations with pre-test activations for each participant. Statistical comparisons were computed at a statistical threshold of *p* < 0.05, corrected for multiple comparisons using Bonferroni correction, and a cluster threshold of 50 voxels.

## Results

The stimulation procedure was well tolerated by all the participants, who did not report any kind of problem during the tDCS administration.

### Cognitive Performance

We conducted a paired samples *T*-test analysis on the DA subtest of the TEA. As expected, we observed no improvements due to the nonspecific control activities which the patients attended between T0 (baseline) and T1 (pre training), either in the experimental (RT: *t* = 1.27; *p* = 0.22; Omission Errors (OE): *t* = 0.52; *p* = 0.61) or in the control group (RT: *t* = 0.45; *p* = 0.66; OEs: *t* = −0.44; *p* = 0.66). Within the experimental group, patients’ performance at T2 (post-training) was significantly better than at T1 (pre-training), with faster RTs (*t* = 3.41; *p* = 0.004) and fewer OEs (*t* = 4.49; *p* < 0.0001), and this improvement was stable 1 month after the end of the treatment (T2 vs. T3 RT: *t* = 0.51; *p* = 0.62; OEs: *t* = 0.20; *p* = 0.84). On the contrary, within the control group, patients’ performance did not increase, either on RT (*t* = −0.006; *p* = 0.99) or on OEs (*t* = 0.92; *p* = 0.37).

At T0 and T3, we administered a series of neuropsychological tests to obtain a precise cognitive profile of the patients before and after the training program. As far as performance on the RBANS is concerned, within the experimental group the analysis did not reveal any statistically significant difference between performance before and after the training on visual-spatial abilities (*t* = −0.670; *p* = 0.256 one-tailed), semantic fluency (*t* = 0.083; *p* = 0.467), working memory (*t* = −0.849; *p* = 0.204), long-term memory (*t* = −1.235; *p* = 0.118); it showed, however, improved performance in the attention task, at the limits of statistical significance (*t* = −1.679; *p* = 0.057). Apathy scores showed significant improvement (*t* = 1.793; *p* = 0.047), while no relevant changes have been detected in the Depression scores (*t* = 0.521; *p* = 0.305).

A split-plot ANOVA with Time as the within subject factor (pre-training computed as the mean between T0 and T1 vs. post-training T2), Type of Group (Control vs. Experimental) as the between subject factor, and age, education level and severity of injury (Glasgow Coma Scale scores) as covariates was performed. It showed no interaction between Time and Age (RT: *F* = 0.54; *p* = 0.47; OEs: *F* = 0.87; *p* = 0.36), or between Time and Level of Education (RT: *F* = 1.55; *p* = 0.22; OEs: *F* = 1.99; *p* = 0.17). As far as the interaction between Time and Severity of injury is concerned, it was statistically significant for OEs (*F* = 4.12; *p* = 0.05), while it was insignificant for RT (*F* = 0.51; *p* = 0.48). As expected, a significant interaction emerged between Time and Type of Group (RT: *F* = 4.13; *p* = 0.05; OEs: *F* = 5.12; *p* = 0.03).

**Figure 2:**
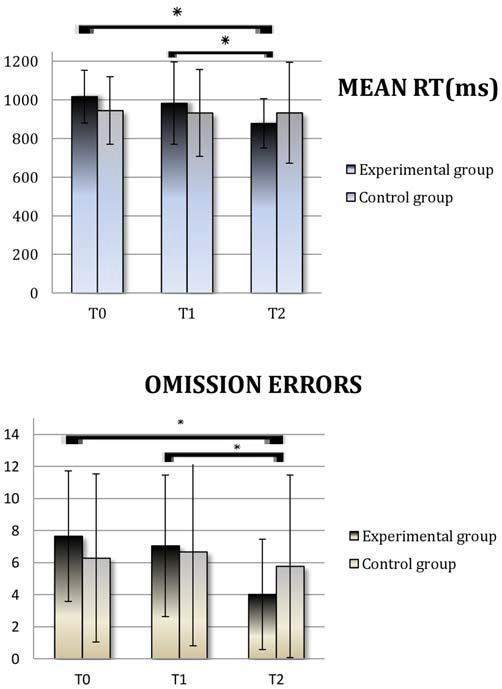
**Divided Attention (DA): Average scores obtained at T0 (Baseline), T1 (pre-Treatment) and T2 (post-Treatment) by the experimental group and the control group.** Reaction time (RT) is expressed in milliseconds. RT; OE, Omission Errors. Asterisks indicate when the difference between performances is statistically significant.

Finally, an independent-sample *t*-test let us exclude that the Type of Montage had an effect on the improvements from pre- to post- training (RT: *t* = 0.098; *p* = 0.923; OE: *t* = 1.218; *p* = 0.243). The main results are represented in Figure 2.

### Brain Activations

**Figure 3:**
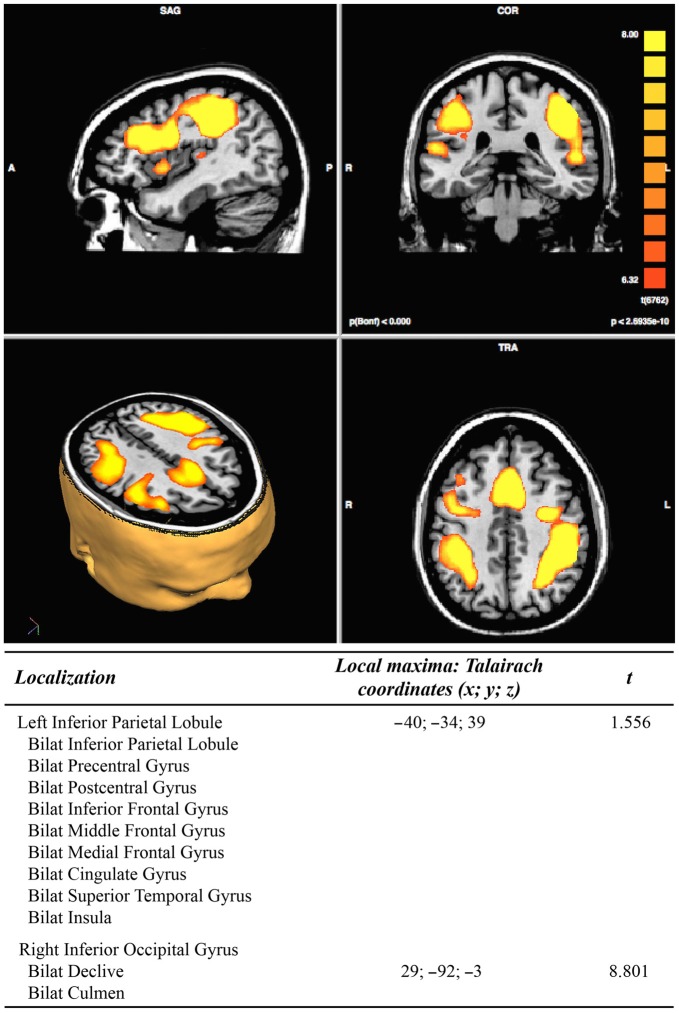
**Results of pre-treatment functional magnetic resonance imaging (fMRI) analysis (*N* = 11; *p* < 2.3965e^−10^)**.

In the pre-treatment, the DA conditions (vs. rest conditions) analysis resulted in two clusters of activation. The first cluster includes extended portions of the following brain areas bilaterally: the inferior parietal lobule, the pre- and post- central gyri, the inferior, middle and medial frontal gyrus, the cingulate gyrus, the superior temporal gyrus, and the insula. The second cluster includes the right inferior occipital gyrus and the cerebellar declive and culmen bilaterally, see Figure 3.

The contrast of the DA conditions vs. the selective attention conditions (auditory + visual) resulted in a cluster of activation including the right posterior cingulate and right lingual gyrus, as well as the cuneus, precuneus and cingulate gyri bilaterally.

**Figure 4:**
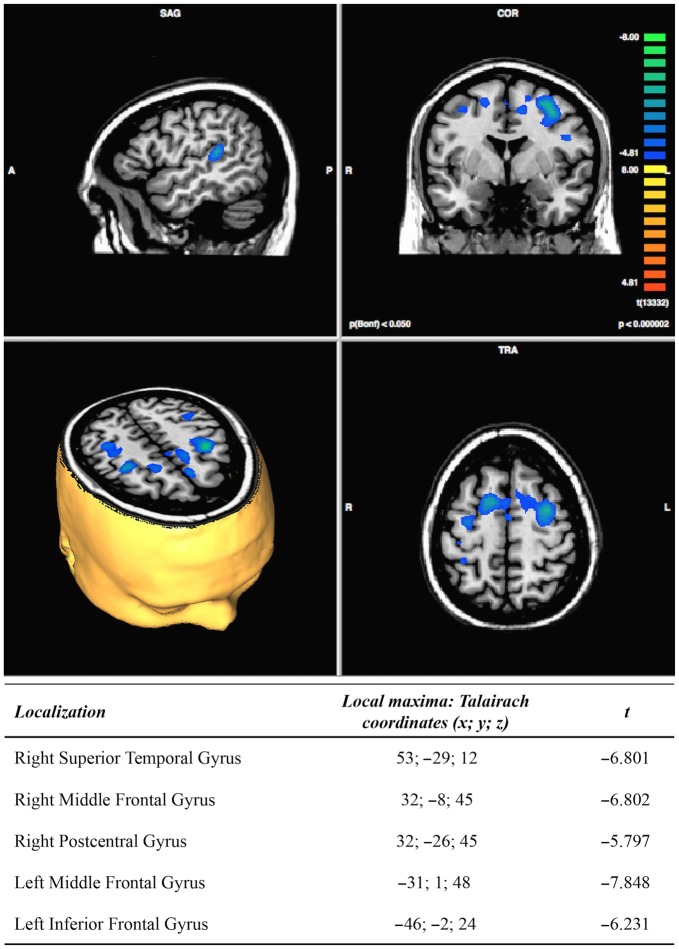
**Results of post-minus pre-treatment fMRI analysis (*N* = 11; *p* < 0.000002)**.

The post vs. pre-treatment comparisons showed a post-treatment significant decrease of activation in the right superior temporal gyrus, the middle frontal gyrus and the postcentral gyrus, as well as in the left middle and inferior frontal gyri, see Figure 4. The contrast of the DA conditions vs. the selective attention conditions, in the post minus pre-treatment comparisons, resulted in a decrease of activation in a small portion of the right cingulate gyrus (Talairach cordinates: 5; −38; 33).

## Discussion

The purpose of this study was to investigate the role of combined tDCS and computer-based training on DA performance following severe TBI. Indeed, given that there is limited evidence of the effectiveness of standard rehabilitation programs aimed at improving attention, especially when chronic patients are involved, this study was intended to explore whether brain stimulation—favoring neuroplastic changes—may enhance the effects of a cognitive training.

Overall, the findings support the idea that 1 week of intensive rehabilitation associated with neuromodulation techniques could significantly contribute to the enhancement of attentional abilities even in chronic patients with a stabilized cognitive profile. Indeed, when performing the DA task, the experimental group obtained significantly better results after the treatment than in the two previous assessments. Furthermore, this improvement was maintained in the follow-up evaluation, performed 1 month after the end of the treatment, highlighting its long-lasting effects. On the contrary, the control group showed no significant improvement. The difference between the experimental and the control group confirmed the efficacy of the combined treatment, as the computerized training alone is not sufficient to produce a significant change on TBI participants’ performance. Among other variables, the severity of the lesion seems to affect the quality, even if not the speed, of performance.

It is worth considering that the improvement concerned attention abilities and was not generalized across other cognitive abilities. Participants belonging to the experimental group showed improved performance in the attention task of the RBANS battery, but not in any other cognitive domain. On this basis, we can conclude that the treatment had specific effects on DA, an outcome which is similar to that reported by Couillet et al. (2010), whose training only ameliorated the ability to complete two tasks simultaneously, without having effects on other cognitive functions.

Some previous works investigated the effect of tDCS over the DLPFC in subacute and chronic TBI patients, but results are not clear-cut. A first pilot study (Kang et al., 2012) explored the effect of a single tDCS session: improvements in RT during an attention task were found immediately post-stimulation, but were not significant after 3 or 24 h. Park et al. (2013) investigated the effectiveness of repeated tDCS sessions associated with a computerized training on attention and memory in a small group of stroke patients, showing post-training improvements of performance. Ulam et al. (2015) studied the cumulative effects of tDCS on electroencephalographic (EEG) oscillations, showing significant modifications correlated with improved performance on attention and working memory tests. On the contrary, the results of another study (Leśniak et al., 2014) did not provide sufficient evidence to support the efficacy of a 15-day rehabilitation training program preceded by tDCS. One possibility is that such contradictory results are due to the utilization of an undifferentiated protocol for all patients, irrespective of the lesion site. Many authors (e.g., Brunoni et al., 2012; Villamar et al., 2012) have pointed out the importance of personalizing the locations of electrodes, especially with brain-damaged subjects. This is particularly relevant when working with TBI patients, given the heterogeneity of their clinical picture and anatomical and functional damage. It is fair to say that some authors (Asloun et al., 2008; Bikson et al., 2010) have claimed that electrode montage has a critical role in modifying the amount of current shunted through the cortex. Indeed, computational modeling studies have predicted that a different position of the return electrode may influence the total current flow produced by the stimulating electrode (Bikson et al., 2010); as a consequence, a change in the return electrode position may influence the current flow under the target region stimulated by the other electrode. However, other modeling studies have shown that, when considering TBI patients, other variables, such as anatomical differences, defects and lesions in the brain—i.e., the presence of cerebrospinal fluid—are expected to distort current flow (Bikson et al., 2012). All these reasons considered, we chose to locate the electrodes according to each participant’s specific damage. This personalized electrode location might have facilitated a more functional network restoration.

Besides the behavioral improvement, our study showed brain modifications: the fMRI assessment seems to suggest a more functional and specific neural organization post-treatment. Indeed, our main fMRI result consists in a decrease in cerebral activations during the DA condition post-treatment. This is in line with the previous literature, reporting significant hyperactivations in patients with TBI performing dual tasks (Rasmussen et al., 2008; Sozda et al., 2011). In particular, Rasmussen et al. (2008) recounted significantly higher activation of a prefrontal-anterior midline-parietal network in TBI subjects in the dual-task condition compared to the matched controls. These regions have been described as being engaged in healthy volunteers as the cost of dual tasking increases (e.g., Herath et al., 2001; Collette et al., 2005). Thus, it seems that, following TBI, there is a functional reorganization within the primary network subserving the task, demonstrating more effortful processing. The authors also claimed that recruitment of these additional cortical resources may be connected to serial rather than parallel processing in low-level dual tasking in TBI patients and thus, that in patients with severe TBI low-level dual task performance may depend on increased attentional and executive guidance. Following this line of reasoning, it is not surprising that the combined tDCS and cognitive training were accompanied by a decrease in cerebral activations: this can be interpreted as normalization of previously abnormal hyperactivations. More specifically, such a decrease concerns a network of areas, including the left DLPFC, the bilateral premotor and supplementary motor areas, the right parietal somatosensory cortex and the right auditory processing cortex. These areas have been found to be involved in dual task processing in healthy subjects (Loose et al., 2003; Nebel et al., 2005; Tachibana et al., 2012), as they subserve the allocation of attention, reflecting the higher executive demand required in divided vs. selective attention tasks. In particular, Johnson et al. (2007) interpreted the increase of activity in DLPFC during DA tasks as reflecting manipulation of information in working memory. According to the authors, when the activity of these areas is temporarily inhibited by slow rTMS, the ability to maintain multiple pieces of information in working memory is also compromised, thus leading to a failure in performing DA tasks.

In conclusion, the proposed treatment resulted in a significant improvement in DA, which was maintained over a long period of time. The combined use of tDCS and computer-based training appears to have favored a neural reorganization, diminishing the patients’ cognitive effort. However, our study presents some limitations. First, the limited number of patients prevented us from performing a subgroup analysis according to the different tDCS electrodes’ montage. Secondly, although patients were in the chronic phase of their injury and their neurological profile was stable, a baseline fMRI test would have helped in better understanding the cerebral mechanisms that accompany the cognitive improvement. Finally, this experiment was aimed at detecting the combined effect of cognitive training and tDCS: future studies involving specific trials aimed at testing the efficacy of the two interventions separately would be very useful in separating the role of each variable.

## Author Contributions

All authors listed have made substantial, direct and intellectual contributions to the work, and approved it for publication.

## Conflict of Interest Statement

The authors declare that the research was conducted in the absence of any commercial or financial relationships that could be construed as a potential conflict of interest.
